# The common *IL1A* single nucleotide polymorphism rs17561 is a hypomorphic mutation that significantly reduces interleukin‐1α release from human blood cells

**DOI:** 10.1111/imm.13584

**Published:** 2022-10-13

**Authors:** Kimberley A. Wiggins, Katerina Pyrillou, Melanie Humphry, Adam S. Butterworth, Murray CH. Clarke

**Affiliations:** ^1^ Section of CardioRespiratory Medicine, Department of Medicine University of Cambridge, The Heart & Lung Research Institute Cambridge UK; ^2^ Dept of Public Health and Primary Care University of Cambridge, The Heart & Lung Research Institute Cambridge UK

**Keywords:** cytokines, genomics, inflammasome, inflammation

## Abstract

Interleukin‐1 alpha (IL‐1α) is a powerful cytokine that drives inflammation and modulates adaptive immunity. Due to these powerful effects, IL‐1α is controlled at multiple levels from transcription to cleavage and release from the cell. Genome‐wide association studies can identify loci that drive important diseases, although often the functional effect of the variant on phenotype remains unknown or small, with most risk variants in non‐coding regions. We find that the common variant rs17561 changes a conserved amino acid in the central region of IL‐1α linking the pro piece to the cytokine domain. Using a recall‐by‐genotype study and whole blood stimulation, we find that minor allele homozygotes release ~50% less IL‐1α than the major allele, with IL‐1β release equivalent. IL‐1α transcript level was identical between groups, implying a post‐transcriptional effect, whilst cleavage of recombinant pro‐IL‐1α by multiple proteases was also equivalent for both forms. Importantly, transfected macrophages also release less minor allele IL‐1α upon inflammasome activation, revealing that reduced secretion is directly caused by the missense amino acid substitution and more minor allele IL‐1α was retained within the cell. Thus, rs17561 represents a very common hypomorphic mutation in IL‐1α. We believe this novel data will be important for determining the potential contribution of IL‐1α to disease and/or physiological processes, for example, by Mendelian randomisation, and may aid patient stratification when considering anti‐IL‐1 therapies.

## Graphical abstract

Interleukin‐1 alpha (IL‐1α) is a powerful cytokine that drives inflammation and modulates adaptive immunity. We investigated whether the common variant rs17561, which changes a conserved amino acid in the central region of IL‐1α, altered cleavage, activation and/or function. Using a recall‐by‐genotype study and whole blood stimulation, we find that minor allele homozygotes release ~50% less IL‐1α than the major allele, with IL‐1β release equivalent.

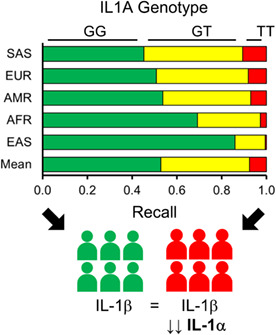

## INTRODUCTION

Inflammation is a key physiological response that protects the host from acute insults such as physical injury or infection. However, many diseases are characterized by and driven by chronic inflammation, including obesity, atherosclerosis, type 2 diabetes and cancer. Interleukin‐1 (IL‐1) is an apical cytokine that modulates both innate and adaptive immunity [1], with the human IL‐1 family containing 11 ligands and 10 receptors that both promote and inhibit inflammation. The principal ligands IL‐1α and β bind the type 1 IL‐1 receptor (IL‐1R1) and induce identical effects [1], including cytokine secretion, adhesion and/or major hostocompatibility complex/Costimulatory molecule expression, vascular leakage [2], Th17 cell differentiation, and enhanced survival and expansion of T cells, even in the presence of Tregs [3]. In addition, autocrine IL‐1α drives the senescence‐associated secretory phenotype (SASP) [4, 5], which mediates many of the deleterious effects of senescent cell accumulation during ageing.

These potent activities of IL‐1 are countered by a receptor antagonist (IL‐1RA), a decoy receptor (IL‐1R2), and expression of IL‐1α and IL‐1β as pro‐proteins that require processing for full biological activity [6, 7, 8, 9]. IL‐1α is canonically cleaved by calpain, which occurs upon necrosis in some cell types and significantly increases activity [9], whilst IL‐1β is typically activated by caspase‐1 after inflammasome engagement [10]. IL‐1α can also be localized in the nucleus due to a nuclear localisation sequence (NLS) within the N‐terminus, which can control release [11]. However, while IL‐1β requires stimulation (e.g., TLR ligation) for expression, IL‐1α is constitutively expressed in many cells.

Single nucleotide polymorphisms (SNPs) are the alteration of a single nucleotide within the genome that is conserved within a population to some appreciable degree. Although SNPs occur most frequently in non‐coding regions, where they might influence transcription, splicing or RNA half‐life, larger functional effects result from SNPs within coding regions. SNPs that change amino acid residues are termed non‐synonymous (nsSNPs) and are very rare due to the evolutionary selection against amino acid substitutions (Shen et al., 2006). Studying human SNPs is important because the genetic variation they create can underlie susceptibility to disease and/or responsiveness to therapy, and in turn reveal new biological mechanisms [12, 13]. The *IL1B* gene has a pLI (probability of loss of function intolerance) score of 0.93, indicating extremely high intolerance to mutations that cause loss of function, and thus very few *IL1B* nsSNPs exist. In contrast, *IL1A* has a pLI score of 0.00 and contains a very common nsSNP (rs17561; MAF 0.27) that results in an alanine to serine substitution in the minor allele form.

rs17561 is reported to be associated with risk for a number of inflammatory diseases and other conditions (Table S1), including systemic sclerosis, breast and ovarian cancer, multiple sclerosis, obesity, CRP level and cardiovascular disease. Whilst association of rs17561 with disease likely indicates that the missense mutation changes IL‐1α function, very few reports have investigated this. Kawaguchi and colleagues examined rs17561 in the context of systemic sclerosis, where they reported increased calpain processing of pro‐IL‐1α in fibroblast lysates derived from minor allele homozygotes also suffering from systemic sclerosis [14]. However, under basal conditions pro‐IL‐1α is complexed to IL‐1R2 in most cells, which protects it from calpain cleavage until cells are activated [9, 15]. Thus, despite the prevalence of rs17561 and its association with important diseases, any functional effect of this nsSNP is currently unknown.

We show that rs17561 changes a highly conserved alanine to a serine in the central region of IL‐1α targeted for activation. Using a recall‐by‐genotype study and whole blood stimulation with lipopolysaccharide (LPS), we find that homozygotes for the minor allele release significantly less IL‐1α than the major allele, with IL‐1β release equivalent, supporting a specific effect on IL‐1α. IL‐1α transcript level after LPS was also identical between groups, implying a post‐transcriptional effect, while cleavage of both forms of IL‐1α by key proteases was identical. Importantly, transfected macrophages also released less minor allele IL‐1α after inflammasome activation, revealing that reduced secretion is directly caused by the amino acid substitution of rs17561, and also more minor allele IL‐1α was retained inside the cell. We believe this novel data will be important for determining the functional contribution of IL‐1α to disease and/or physiological processes.

## MATERIALS AND METHODS

All material from Sigma‐Aldrich unless otherwise stated.

### Study design

Cohorts were selected from individuals already recruited into the Cambridge BioResource (CBR) that were genotyped using MS Chip, Exome Chip and Core Exome Chip (Illumina), supplemented with custom TaqMan SNP genotyping assays (Applied Biosystems). At the time of the study, the CBR cohort had ~6000 individuals genotyped for rs17561 with a MAF of 0.269 which equates to 6.7% or 402 individuals homozygous for the minor allele. Pre‐defined inclusion criteria were: male only; age 18–55; BMI ≥18.5 and ≤ 32; no inflammatory conditions (e.g., psoriasis, rheumatoid arthritis, gout, IBD, etc); no infections (e.g., HepB, HepC, HIV, etc); no vaccination within the last three months; no self‐medication with anti‐inflammatory agents that day (e.g., paracetamol, aspirin, ibuprofen, etc); no steroids that week; no medication for allergies or high blood pressure; no statins. Blood was taken with informed consent and full ethical approval from the National Research Ethics Service by CBR research nurses using an 18G butterfly needle and passed directly to the researcher. Participants remained anonymous and genotypes remained blinded until the end of the study. Initial pilot studies establishing the whole blood stimulation system provided variability of IL‐1α release to enable a power calculation (OpenEpi online calculator). This showed that 23 individuals were needed for 95% confidence (2‐sided) at 80% power with a 1:1 group size ratio. Thus, 25 individuals each for the major and minor rs17561 homozygotes were recruited, with one minor allele individual subsequently excluded for taking prescribed aspirin on alternate days.

### Whole blood LPS stimulation

Blood was drawn into EDTA for full blood counts (Addenbrooke's Clinical Biochemistry autoanalyser) or lithium heparin for whole blood stimulation assays, as this best preserves cell function [16]. Whole blood stimulation was conducted in triplicate with blood diluted 1:2, or as indicated, into a 6‐well plate with Iscove's Modified Eagle media (10 U/mL penicillin, 10 mg/mL streptomycin, 5 mg/mL L‐glutamine), with or without LPS (1 μg/mL, or as indicated; Sigma, L2630). Sufficient media ± LPS was batch mixed and frozen (−80°C) before the study to ensure consistency. Where indicated, whole blood was preincubated (30 mins) with zVAD‐fmk (20 μM; Enzo), VX‐765 (10 μM) or MCC950 (20 μM; both InvivoGen) before LPS. Diluted blood was incubated (22 h, 37°C), collected, centrifuged (400 g, 5 mins) and the clarified supernatant from individual triplicates frozen (−80°C). Remaining adherent cells were harvested into TRIzol (Invitrogen), pooling triplicate wells, and stored (−80°C).

### Cytokine and LDH detection

Cytokines were measured using IL‐1α, IL‐1β and IL‐6 bead ELISAs (Life Technologies) analysed with a flow cytometer (Accuri C6), while IL‐1R2 was quantified with a plate ELISA (R&D), all as per the manufacturer's instructions. LDH was measured with the Pierce LDH Cytotoxicity Assay Kit (ThermoFisher), as per the manufacturer's instructions.

### Recombinant protein expression, purification and cleavage

pro‐IL‐1α cDNA was cloned into pGEX‐4 T‐3 (GE), with the minor allele mutation introduced by site‐directed mutagenesis. For bacterial expression, transformed Rosetta cultures were IPTG‐induced, incubated (4 h, room temp), harvested by centrifugation (5000 g, 15 min, 4 °C), lysed (PBS, 1x; DTT, 1 mM; EDTA, 10 mM; benzonase, 30 U/mL; lysozyme, 55 k U/g [both Novagen]; protease inhibitor cocktail) with sonication, and clarified by centrifugation (5525 g, 1 h, 4°C). Filtered supernatants (0.45 μm) were loaded onto a GSTrap column (GE), washed (PBS; DTT, 1 mM; EDTA, 1 mM) and eluted (Tris, 50 mM; NaCl, 100 mM; DTT, 1 mM; reduced L‐Glutathione, 50 mM; pH 8) using an ÅKTA FPLC system (GE). Eluted protein was dialysed overnight (Tris, 10 mM; NaCl, 50 mM; pH 8) and stored (−80°C) in glycerol (10%). pro‐IL‐1α protein concentration was normalized by SDS PAGE, coomassie staining (G‐250; Biorad) and quantitative imaging (Odyssey, LI‐COR). Recombinant pro‐IL‐1α (4 μg/mL) was incubated with active proteases (units as indicated) in specific reaction buffers: calpain (1 h, RT; Calbiochem; [NaCl, 100 mM; CaCl_2_, 2 mM; DTT 1 mM]); thrombin (2 h, RT; Novagen; [Tris–HCl, 20 mM, pH 8.4; NaCl, 150 mM; CaCl_2_, 2.5 mM]); elastase (2 h, RT; Enzo; [Tris, 50 mM, pH 8.8]); cathepsin B (1 h, 40°C; Enzo; [Na_3_PO_4_, 10 mM, pH 6; EDTA, 1.3 mM; DTT, 1 mM]).

### IL‐1 bioassay

HeLa cells (ECACC) were cultured in DMEM with penicillin, streptomycin, L‐glutamine and 10% FCS and passaged at ~80% confluency. HeLa cells were plated (1 × 10^5^/well; 48‐well) and allowed to adhere (16 h, 37°C). Media was refreshed and cells were incubated (6 h, 37°C) ± test samples (e.g., cleavage assay products). Supernatant was collected and clarified (12 000 g, 3 mins), before storage (−80°C). Specific IL‐1α activity was inferred via inhibition of IL‐6 production by an IL‐1α neutralizing antibody (2 μg/mL; PeproTech). For IL‐1R2/IL‐1RAcP antagonism, A^114^ or S^114^ pro‐IL‐1α (4 μg/mL) was preincubated (20 mins) with IL‐1R2 (R&D) and/or IL‐1RAcP (Sino Biological) at the indicated concentration before addition to HeLa cells, as above. Every experiment contained a media‐only negative control and an IL‐1α (10 ng/mL; PeproTech) positive control.

### Gene expression analysis

RNA was extracted using TRIzol, as per the manufacturer's instructions. Briefly, cells were lysed in TRIzol (5 mins, RT), chloroform added, vigorously shaken, and incubated (3 mins, RT). Samples were centrifuged (12 000 g, 15 mins, 4°C), the upper aqueous phase mixed with isopropanol (100%), incubated (10 mins, RT), and centrifuged (12 000 g, 10 mins, 4°C) to pellet RNA. The RNA pellet was washed with ethanol (75%), centrifuged (7500 g, 5 mins, 4°C), and air dried before resuspension in water. RNA was quantified using a Nanodrop and stored (−80°C). cDNA was prepared using the SuperScript VILO cDNA synthesis kit (ThermoFisher), as per the manufacturer's instructions. 0.5 μl of each sample was pooled for the standard curve, and samples were stored at −20°C. Each qPCR reaction contained H_2_O (14.3 μl), 10x Buffer (2 μl), MgCl_2_ (25 mM; 1.6 μl), 0.1 μl AmpliTaq Gold (0.1 μl), Taqman Probe (0.5 μl) (all ThermoFisher), dNTPs (10 mM each; 0.4 μl), and cDNA (1 μl). qPCR used a thermocycler (Rotorgene), and analysis was via a standard curve with cDNA generated from RNA pooled from every sample, along with *TBP* and *GUSB* reference genes.

### Monocyte depletion and macrophage transfection

Monocytes were immunodepleted from diluted whole blood by incubation with anti‐CD14 beads (8 μg/mL, 10 mins; Biolegend) and magnetic separation using Dynabeads Biotin Binder (25 μl; Invitrogen). Immortalized mouse bone marrow‐derived macrophages (iBMDMs) were cultured in RPMI with penicillin, streptomycin, L‐glutamine and 10% FCS and passaged at ~80% confluency. iBMDMs were transfected with empty pcDNA3.1, or pcDNA3.1 containing full‐length human *IL1A* ^A114^ *or IL1A* ^S114^ by nucleofection (Lonza). 24 h after transfection iBMDMs were LPS treated (1 μg/mL, 4 h), followed by nigericin (15 μM, 1 h; Invivogen), digitonin (Promega) or saponin (both 10 μg/mL, 1 h). Adherent cells were lysed (0.5% NP‐40, 5 mM EDTA, 5 mM EGTA, 100 μM calpeptin (Enzo), 1x protease inhibitor cocktail (Calbiochem) in PBS) for 20 mins on ice. Collected conditioned media and lysates were clarified and stored (−20°C). For analysis of IL‐1α expression by western, cells were collected after LPS treatment only.

### Western blotting

Westerns were performed as previously described with lysis of cells directly in Laemmli buffer, SDS‐PAGE, transfer onto PVDF, blocking (5% milk), incubation (16 h, 4°C) with rabbit anti‐IL‐1α pAb (1:500; Peprotech), before washing (PBS/Tween) and incubation (1 h, RT) with anti‐rabbit HRP (1:2000; GE). After washing, membranes were visualized with ECL reagent (Amersham) and X‐ray film (Fujifilm). Coomassie staining was used as a loading control.

### Statistical analysis

All statistical analyses were carried out using Prism 7 (Graphpad). Before statistical testing for significance, data were analysed with a Kolmogorov–Smirnov test, which reported a normal distribution.

Data were subsequently analysed by unpaired *t*‐test (two‐tailed), one‐way ANOVA with Dunnett's post hoc or one‐way ANOVA with Tukey's posthoc. IL‐1α/IL‐1R2 correlation used Pearson's. *n* = an independent biological replicate performed in duplicate or triplicate – never a technical replicate.

## RESULTS

### rs17561 changes a conserved amino acid in the region of IL‐1α targeted for activation

**FIGURE 1:**
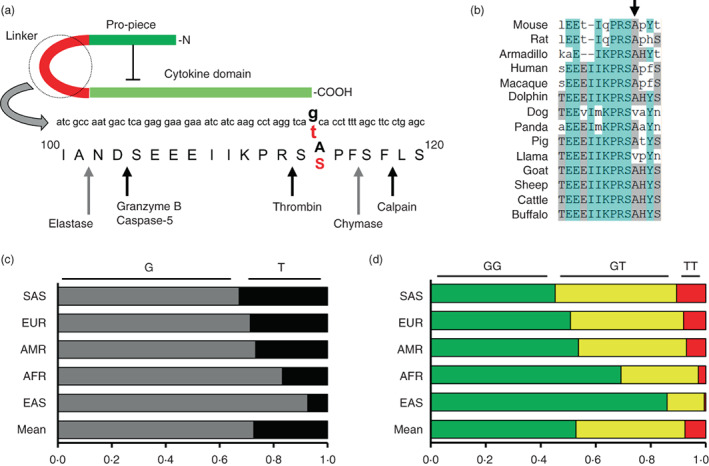
rs17561 changes a conserved amino acid in the region of IL‐1α targeted for activation. (a) Schematic showing the domain structure of pro‐IL‐1α, the linker region targeted by proteases that activate pro‐IL‐1α, and the coding sequence changes of rs17561. Black/red bold text indicates major/minor allele coding, respectively. Black arrow = known protease site; grey arrow = predicted protease site. (b) Multi‐species alignment of IL‐1α protein sequences showing high conservation of the alanine changed by rs17561 (arrow). (c, d) frequency of rs17561 alleles (c) and genotypes (d) in super population groups. AFR = African; AMR = admixed American; EAS = east Asian; EUR = European; SAS = south Asian.

rs17561 is a common nsSNP that is reported to be associated with the risk of many diseases (Table S1) and traits (Table S2), but if this coding change affects IL‐1α function is currently unknown. We find rs17561 results in an amino acid substitution within the linker region of IL‐1α that is targeted by proteases for cytokine maturation and full activity (Figure 1a) [8, 9, 15, 17, 18]. The minor allele form of rs17561 has alanine substituted for serine at amino acid 114, and thus a change from a non‐polar hydrophobic amino acid to a polar uncharged. Furthermore, Ala^114^ is highly conserved between disparate species (Figure 1b) with 85% containing Ala^114^ and 13.4% containing the conservative substitution of Val^114^ (Figure S1). Prediction software reports the SNP as ‘deleterious’ (SIFT) and ‘probably damaging’ (PolyPhen‐2). However, rs17561 is surprisingly common for an nsSNP, with minor allele frequencies (MAFs) from 33% in South Asians to 7.3% in East Asian super populations (Figure 1c), and resultant frequencies of minor allele homozygotes from 10.7% to 0.5% (Figure 1d). Thus, rs17561 changes a conserved amino acid within a key functional region of IL‐1α, which may explain its effect on disease.

### Validation and process for the recall‐by‐genotype whole blood stimulation study

**FIGURE 2:**
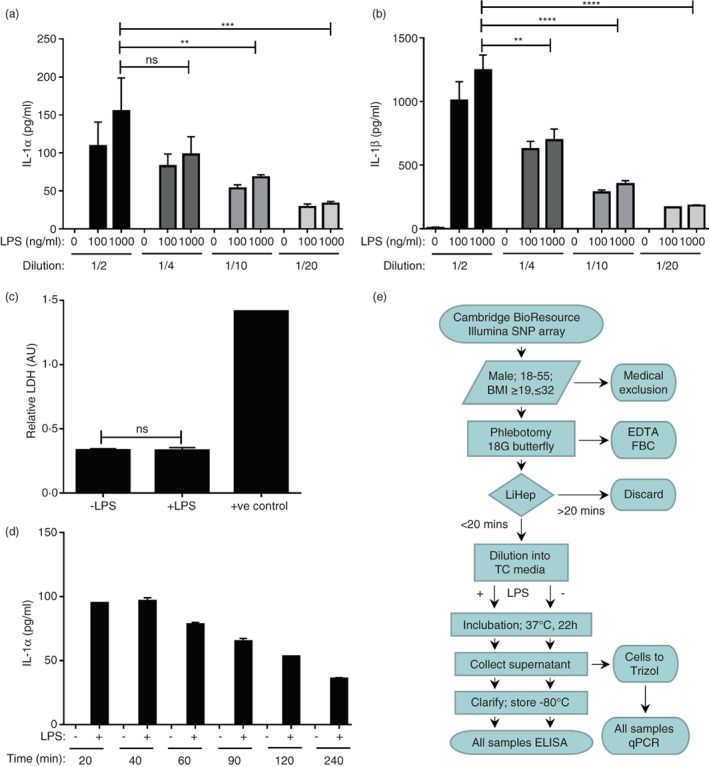
Validation and process for the recall‐by‐genotype whole blood stimulation study. (a, b) ELISA data showing the level of IL‐1α (a) and IL‐1β (b) in the clarified supernatant of whole blood diluted and treated ± LPS for 22 h, as indicated. (c) Spectrophotometric assay reporting the level of lactate dehydrogenase (LDH) in diluted whole blood after treatment ± LPS. (d) ELISA data showing the level of IL‐1α in the clarified supernatant of whole blood diluted and treated ± LPS at the time indicated after blood collection. (e) Process flowchart outlining the study design. See materials and methods for inclusion and exclusion criteria. FBC = full blood count; LiHep = lithium heparin; TC = tissue culture. Data represent mean ± SEM of n = 4 (a, b), 8 (c), 2 (d); *p* = ** ≤ 0.01, *** ≤ 0.001, **** ≤ 0.0001; ns = not significant.

To identify any gross effects of rs17561 on IL‐1α function we utilized the Cambridge NIHR BioResource to conduct a recall‐by‐genotype study measuring IL‐1α release in a physiological cell system. To remove the variability generated by the purification of specific cell types (e.g., PBMCs via density gradient), we utilized whole blood stimulation [16, 19]. This also has the advantage of maintaining all blood cell types together in a plasma matrix, which provides a more physiological milieu for cell activation. Dilution of whole blood with media, followed by LPS stimulation and subsequent culture resulted in the release of IL‐1α (Figure 2a) and IL‐1β (Figure 2b) at levels readily quantifiable by ELISA. Notably, no cytokines were detected without LPS stimulation. Dilution of whole blood four‐fold maintained similar levels of cytokine release, while 100 ng/mL of LPS gave lower cytokine levels than 1 μg/mL of LPS. To maximize assay sensitivity, a two‐fold blood dilution and 1 μg/mL of LPS were subsequently used. As IL‐1α can be secreted physiologically [20, 21, 22] or released after necrosis and/or loss of plasma membrane integrity [9, 23] we evaluated cell death during culture. Importantly, LDH level in culture supernatants was equivalent with or without LPS (Figure 2c), indicating that IL‐1α or IL‐1β release had not occurred via cell death. Finally, we tested the impact of time between phlebotomy and blood processing/culturing. This revealed a huge effect of time, with intervals over 40 mins leading to a linear decrease in cytokine release (Figure 2d). Thus, we conducted all further blood donations on site, with blood processed and cultured within 20 mins of phlebotomy. As leucocytes are the main source of IL‐1α and IL‐1β, and haematocrit directly affects cytokine concentration in a given volume of blood, we also performed full blood counts upon donation. Finally, after the collection of supernatant from cultured blood, cells were extracted into TRIzol for future RNA purification. A process flowchart outlining the entire study design is shown (Figure 2e).

### Whole blood from rs17561 minor allele homozygotes releases significantly less IL‐1α

**FIGURE 3:**
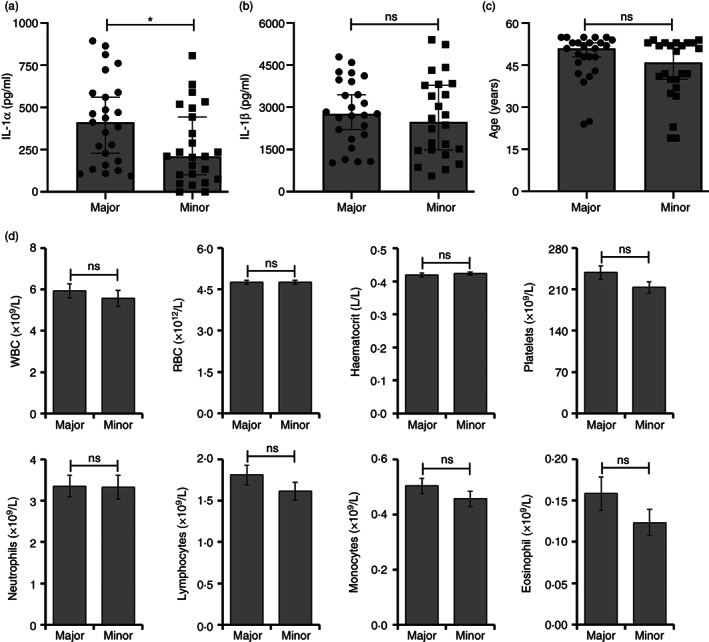
Whole blood from rs17561 minor allele homozygotes releases significantly less IL‐1α. (a, b) ELISA data showing the level of IL‐1α (a) and IL‐1β (b) in the clarified supernatant of whole blood from rs17561 major/minor homozygote individuals after dilution and treatment ± LPS for 22 h. (c) Age range of rs17561 major/minor homozygote individuals donating blood after recall‐by‐genotype. (d) Full blood count data from rs17561 major/minor homozygote individuals at the time of donation. RBC = red blood cells; WBC = white blood cells. Data represent median ± 95% CI (a–c), or mean ± SEM of n = 25 (major), 24 (minor); *p* = * ≤ 0.034; ns = not significant.

Using this recall‐by‐genotype study design we recruited eligible individuals homozygous for the rs17561 major or minor allele. Testing all samples for cytokine production together by ELISA revealed significantly less IL‐1α release from minor allele carriers compared to major (Figure 3a), but no difference in IL‐1β release between groups (Figure 3b). Importantly, no significant difference in the age of recruited individuals was found between genotypes (Figure 3c) and no significant changes in full blood count parameters that could explain the difference in IL‐1α level were found (Figure 3d). Similarly, normalization of IL‐1α or IL‐1β level to blood cell counts within each individual maintained and/or lowered the significant p‐value for IL‐1α, but increased the non‐significant p‐value for IL‐1β (Table 1), suggesting the variation in IL‐1β levels across individuals relates to blood cell counts, but the lower IL‐1α release seen in minor allele homozygotes is not. Together this suggests that the rs17561 minor allele variant specifically results in less secretion of IL‐1α after physiological stimulation of whole blood, with no effect on IL‐1β release.

**TABLE 1:** Normalization of IL‐1α and IL‐1β level to blood cell count. Fold change of means and *p*‐value for IL‐1α and IL‐1β release between major and minor alleles before (raw) and after normalization to blood cell counts within a given individual

Parameter	IL‐1α		IL‐1β	
	Fold	*p*‐value	Fold	*p*‐value
Raw	0.643	**0.034**	0.926	0.577
WBC	0.649	**0.048**	1.033	0.823
Haematocrit	0.646	**0.037**	0.910	0.491
Platelets	0.674	0.084	0.923	0.627
Neutrophils	0.619	**0.031**	0.973	0.858
Lymphocytes	0.710	0.133	1.107	0.533
Monocytes	0.636	0.062	1.035	0.833
Eosinophils	0.683	0.266	1.061	0.848

### Less IL‐1α from rs17561 minor allele homozygotes is not due to increased IL‐1R2 binding reducing detection

**FIGURE 4:**
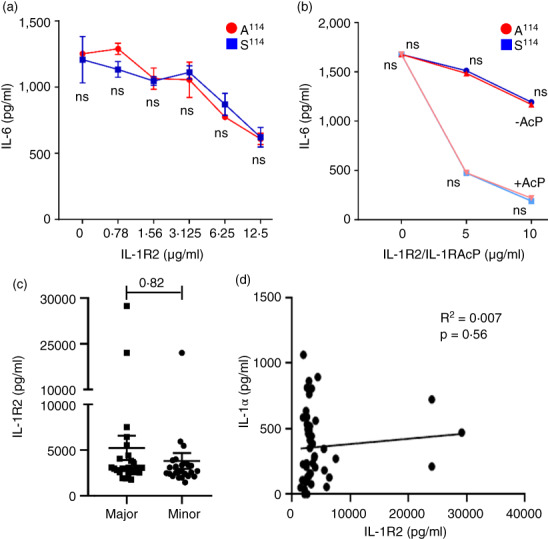
Less IL‐1α from rs17561 minor allele homozygotes is not due to increased IL‐1R2 binding reducing detection. (a, b) ELISA data showing IL‐1α‐dependent IL‐6 production by HeLa cells incubated with recombinant IL‐1α equivalent to major (a^114^) or minor (s^114^) allele forms, along with increasing concentrations of IL‐1R2 (a), or IL‐1R2 and IL‐1RAcP (b). (c) ELISA data showing the level of IL‐1R2 in the clarified supernatant of diluted whole blood from rs17561 major and minor homozygote individuals treated without LPS for 22 h. (d) Pearson's correlation between released IL‐1α and IL‐1R2 level in rs17561 major and minor homozygotes. Data represent mean ± SEM of *n* = 2 (a, b), 25 (major), 24 (minor) (c, d); *p* = as indicated; ns = not significant.

IL‐1α and IL‐1β can be antagonized by binding to the decoy receptor IL‐1R2 [24]. IL‐1R2 is at high levels in the circulation along with the co‐receptor IL‐1RAcP that increases IL‐1 binding affinity [25]. Thus, if rs17561 alleles altered the interaction between IL‐1α and IL‐1R2 it could potentially interfere with IL‐1α detection by ELISA, or affect proteolytic cleavage and secretion of IL‐1α [9]. To investigate this, we used a simple bioassay of IL‐1α‐dependent IL‐6 production from HeLa cells. Increasing concentrations of IL‐1R2 led to equivalent antagonism (reduced IL‐6 production) of both major A^114^ and minor S^114^ forms of recombinant IL‐1α (Figure 4a). Similarly, addition of IL‐1RAcP along with IL‐1R2 also led to an identical increase in antagonism of either form of IL‐1α (Figure 4b). In addition, we also tested the level of soluble IL‐1R2 in diluted blood samples from all individuals, which showed no difference between genotypes (Figure 4c) and no correlation between IL‐1α and IL‐1R2 levels (Figure 4d). Finally, there was also no intrinsic difference in the ability of the IL‐1α ELISA to detect cleaved recombinant forms of A^114^ or S^114^ pro‐IL‐1α (See Figure 6a, c, e, g below). Together, this suggests that rs17561 variants do not alter the interaction between pro‐IL‐1α and IL‐1R2/IL‐1RAcP, and that interference with IL‐1α detection by IL‐1R2, or an altered affinity of the ELISA for A^114^ versus S^114^ IL‐1α, cannot explain the decreased IL‐1α levels released by minor allele homozygotes (Figure 3a).

### *IL1A* expression is unaffected by rs17561 alleles

**FIGURE 5:**
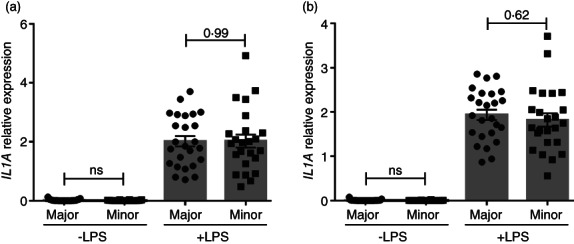
*IL1A* expression is unaffected by rs17561 alleles. (a, b) qPCR data showing the level of *IL1A* (a) and *IL1B* (b) transcript in blood cells from major/minor rs17561 individuals after whole blood dilution and treatment ± LPS for 22 h. Data represent mean ± SEM of *n* = 25 (major), 24 (minor); *p* = as indicated; ns = not significant.

rs17561 is in linkage disequilibrium with rs1800587, another IL‐1α SNP within the promoter region of *IL1A*. Thus, if rs1800587 altered *IL1A* expression this could potentially explain the reduced IL‐1α secretion from the rs17561 minor allele homozygotes (Figure 3a), independent of the amino acid substitution. However, qPCR analysis of the transcript revealed identical LPS‐induced *IL1A* expression between genotypes (Figure 5a), with a *p*‐value of 0.99. Again, rs17561 genotype had no effect on *IL1B* expression (Figure 5b). This shows that the reduced secretion of IL‐1α from rs17561 minor allele homozygote blood cells is not via reduced *IL1A* expression, and thus must be governed post‐transcriptionally.

### pro‐IL‐1α cleavage by key proteases is equivalent between rs17561 alleles

**FIGURE 6:**
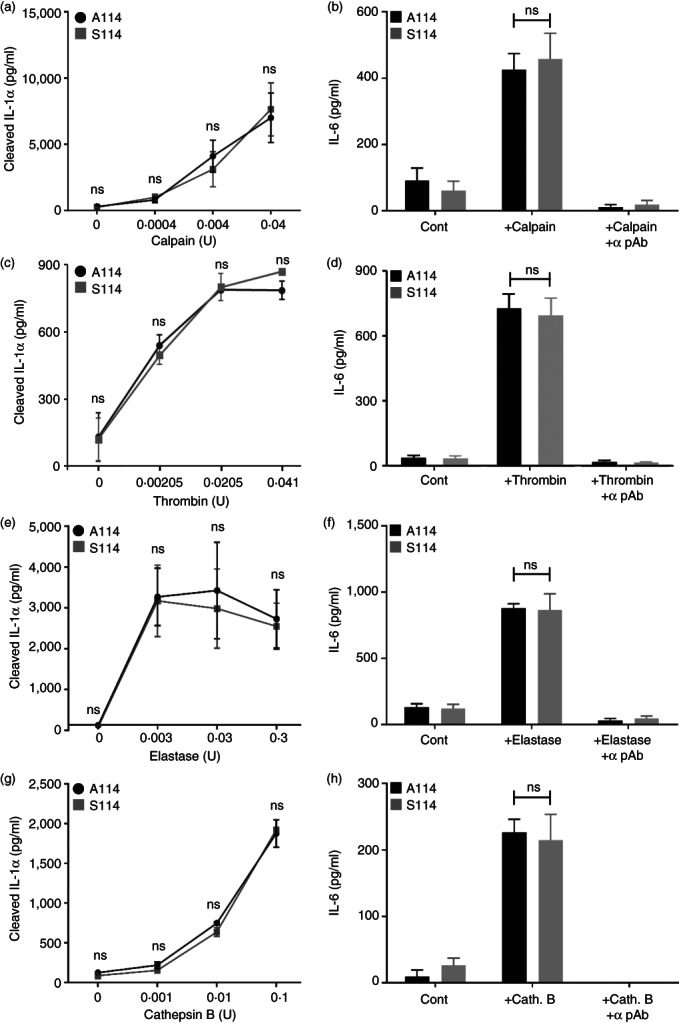
Pro‐IL‐1α cleavage by key proteases is equivalent between rs17561 alleles. (a–h) recombinant pro‐IL‐1α equivalent to major (a^114^) or minor (S^114^) allele forms was incubated with calpain (a, b), thrombin (c, d), elastase (e, f) or cathepsin B (g, h), and reaction products assayed with a cleaved IL‐1α‐specific ELISA to determine the level of IL‐1α cleavage (a, c, e, g), or cytokine activity measured through IL‐1‐dependent IL‐6 production by HeLa cells, ± neutralizing IL‐1α antibody (+α pAb) (b, d, f, h). Data represent mean ± SEM of *n* = 3 (individual protein purifications and assays); ns = not significant.

rs17561 variants result in an amino acid change in the region of IL‐1α targeted by proteases (Figure 1a). As minor allele homozygotes release less IL‐1α (Figure 3a), we compared the cleavage of recombinant A^114^ and S^114^ pro‐IL‐1α by a range of proteases and the subsequent cytokine activity generated. Calpain [26], thrombin [17] and elastase [8] are shown to cleave pro‐IL‐1α, while cathepsin B is reported to cleave pro‐IL‐1β [27]. Both forms of pro‐IL‐1α were found to be cleaved equivalently by all four proteases at a range of concentrations (Figure 6a, c, e, g), and this processing translated to equivalent levels of cytokine activity (Figure 6b, d, f, h). Thus, despite the proximity of the amino acid substitution to the protease sites, this data suggests that reduced cleavage of S^114^ pro‐IL‐1α by these proteases is unlikely to mediate the reduced IL‐1α secretion seen in minor allele homozygotes (Figure 3a).

### Monocyte‐derived IL‐1α from both rs17561 alleles is equally dependent on caspases or inflammasomes

**FIGURE 7:**
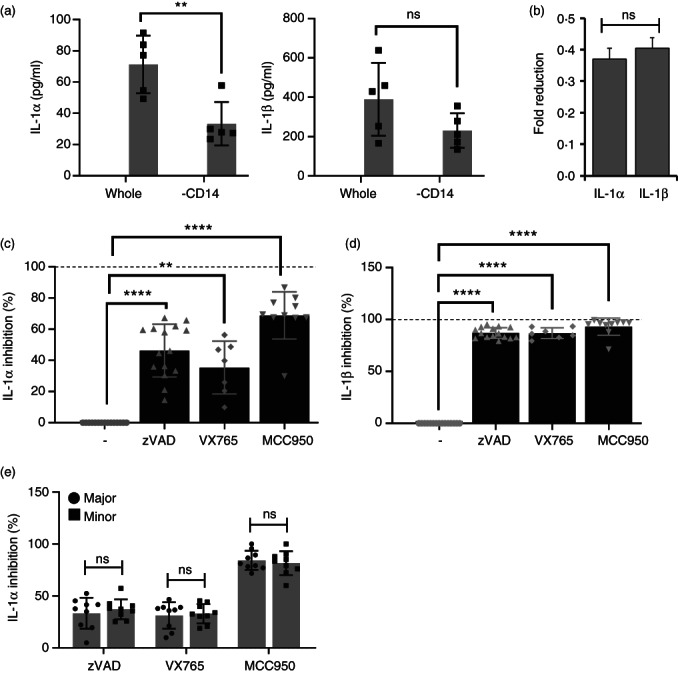
Monocyte‐derived IL‐1α from both rs17561 alleles is equally dependent on caspases or inflammasomes. (a) ELISA data showing the level of IL‐1α and IL‐1β in diluted whole blood or CD14 immunodepleted blood following LPS treatment. (b) Fold reduction in IL‐1α and IL‐1β level after CD14 immunodepletion. (c, d) ELISA data showing inhibition of IL‐1α (c) and IL‐1β (d) release from diluted whole blood pretreated ± caspase inhibitors (zVAD, VX765) or ± NLRP3 inflammasome inhibitor (MCC950) before LPS treatment. (e) ELISA data showing inhibition of IL‐1α release into the clarified supernatant of major and minor allele whole blood after dilution and pre‐treatment ± caspase inhibitors or an NLRP3 inflammasome inhibitor, before LPS treatment for 22 h. Data represent mean ± SEM of *n* = as shown; *p* = ** ≤ 0.01, **** ≤ 0.0001; ns = not significant.

To help understand at which stage less IL‐1α is released by rs17561 minor allele homozygotes, we first determined which blood cells release IL‐1. Immunodepletion of monocytes with anti‐CD14 beads reduced IL‐1 production (Figure 7a) in line with the level of monocyte depletion (69 ± 9%), and reduced production was the same for IL‐1α and IL‐1β (Figure 7b). This implies that monocytes are the predominant producer of both cytokines. Monocyte IL‐1β release is known to require inflammasomes and caspases [10]. However, IL‐1α secretion is still not understood. Thus, we pre‐incubated diluted whole blood with caspase inhibitors (zVAD, pan‐caspase; VX‐765, non‐peptide caspase‐1‐specific) or an NLRP3 inflammasome inhibitor (MCC950) before LPS stimulation and culture. Interestingly, IL‐1α release was only partially inhibited by zVAD, VX‐765 and MCC950 (Figure 7c), while IL‐1β release was near totally inhibited (Figure 7d). This suggests that IL‐1α release is only partially inflammasome/caspase‐dependent, while IL‐1β release is entirely inflammasome/caspase‐dependent. Thus, IL‐1α release in whole blood might occur via two distinct mechanisms, with reduced IL‐1α from rs17561 minor alleles (Figure 3a) due to an inability of S^114^ IL‐1α to be released via one of these pathways. To investigate this we recalled major and minor homozygotes and treated their diluted whole blood ± zVAD, VX‐765 or MCC950 and compared LPS‐induced IL‐1α production within individuals. However, inhibition of IL‐1α release was equivalent between both groups (Figure 7e), suggesting lower IL‐1α release in minor allele homozygotes is not due to a differential dependence on caspases or inflammasomes.

### Significantly less S^114^ IL‐1α is also released from macrophages after inflammasome activation

**FIGURE 8:**
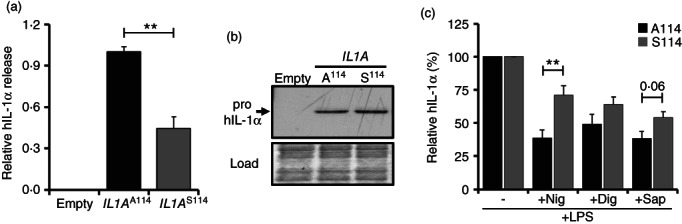
Significantly less S^114^ IL‐1α is released from macrophages after inflammasome activation. (a) ELISA data showing the relative release of human IL‐1α (hIL‐1α) from immortalized murine bone marrow‐derived macrophages (iBMDMs) after transfection of empty pcDNA3.1, or pcDNA3.1 containing full‐length human *IL1A* ^A114^ *or IL1A* ^S114^, followed by activation with LPS/Nigericin. (b) Western blot for hIL‐1α showing the expression level of human a^114^ and S^114^ pro‐IL‐1α after transfection into iBMDMs. (c) ELISA data showing relative level of hIL‐1α retained within immortalized murine bone marrow‐derived macrophages (iBMDMs) after transfection of empty pcDNA3.1, or pcDNA3.1 containing full‐length human *IL1A* ^A114^ *or IL1A* ^S114^, followed by LPS stimulation and subsequent treatment with nigericin (+Nig), digitonin (+dig) or saponin (+sap). Data represent mean ± SEM of *n* = 3 (a), 4 (c), representative of 2 (b); *p* = ** ≤ 0.01, or as indicated; ns = not significant.

To examine the effects of rs17561 in a tractable cell system we expressed full‐length A^114^ and S^114^ human *IL1A* in murine macrophages, activated inflammasomes with LPS/nigericin and specifically measured the release of human IL‐1α. Interestingly, ~50% less IL‐1α was released from cells transfected with S^114^ expressing vectors compared to A^114^ (Figure 8a). Importantly, A^114^ and S^114^ pro‐IL‐1α were expressed at equivalent protein levels (Figure 8b), indicating that reduced release of the S^114^ form was not due to lower expression. Importantly, this shows that lower S^114^ IL‐1α release also occurs in macrophages and is not limited to monocytes in the whole blood system (Figure 7a, b), and again this excludes a serum factor or other artefact reducing IL‐1α detection in minor allele individuals. To test if the reduced S^114^ IL‐1α release was due to it somehow being retained within the cell after inflammasome activation, we compared the amount of A^114^ and S^114^ IL‐1α inside the cell after LPS/nigericin treatment. This revealed that nearly twice as much S^114^ IL‐1α was still in the cell compared to A^114^ (Figure 8c). Interestingly, a trend for more S^114^ IL‐1α left within the cell was also seen after treatment with the pore‐forming detergents digitonin and saponin (Figure 8c), suggesting that retainment of S^114^ IL‐1α was not due to an inability of it to pass through gasdermin D pores formed after inflammasome activation. Together, this proves that the reduced release of IL‐1α from minor allele homozygotes (Figure 3a) is directly due to the missense amino acid substitution of the rs17561 variant and likely caused by increased binding of S^114^ IL‐1α to factors within the cell.

## DISCUSSION

Genetic and genome‐wide association studies have enhanced our understanding of many complex inflammatory diseases (e.g., NOD2 variants in Crohn's disease). However, in many cases, the functional consequence that links the risk variant to disease pathogenesis has remained unknown. This is often because most risk variants are in non‐coding regions of the genome that can act over long distances. Numerous immune mediators show few variants due to a very high intolerance for loss‐of‐function (LOF) mutations (e.g., Foxp3; pLI score 0.99), while many other identified risk variants associate with intracellular proteins, which are hard to target. Cytokines are key signalling intermediaries of the immune system that often drive inflammatory diseases, and represent good therapeutic options due to their extracellular action via cell surface receptors, thereby providing two targets. Thus, discovering LOF or hypomorphic variants in cytokines is informative as it provides data somewhat analogous to a clinical trial utilizing an inhibitor, with both resulting in decreased protein function. Indeed, drug targets that are supported by human genetic data are twice as likely to result in a positive clinical trial outcome [28].

Here we report that the IL‐1α variant rs17561 results in the substitution of a conserved amino acid residue within the central region of IL‐1α that is targeted for cytokine activation. rs17561 minor allele homozygotes released significantly less (~50%) IL‐1α from LPS stimulated whole blood, with IL‐1β levels unaffected. Multiple lines of evidence exclude reduced IL‐1α detection as a possible explanation for the lower levels seen with the minor allele homozygotes. Furthermore, identical *IL1A* transcript levels are found between rs17561 alleles, implying that decreased IL‐1α release is due to a post‐transcriptional mechanism. However, despite proximity of the substitution to known protease sites, recombinant major and minor allele forms of IL‐1α are processed equivalently by key proteases. Importantly, minor allele IL‐1α expressed in macrophages is also released less after inflammasome activation, demonstrating that lower secretion is intrinsic to the amino acid substitution. Together, this reveals that the rs17561 minor allele effectively represents a hypomorphic mutation in IL‐1α that will be of use to identify or understand diseases and/or physiological processes specifically dependent on IL‐1α.

Recall‐by‐genotype studies are a useful tool to help identify any biochemical effect of a genetic variant. However, as many variants have only modest effects on disease risk, detecting a quantifiable change is difficult and often lost within the intrinsic variability of a given assay. Human immune responses are typically measured ex‐vivo in PBMCs after isolation by density gradient centrifugation, and sometimes after cryopreservation to allow batch testing. However, every analytical step post‐phlebotomy introduces variability that cannot be controlled for, and thus reduces the power to see the effect. By using whole blood stimulation assays with a single batch of pre‐mixed culture media, the only technical variability of our assay is pipetting accuracy, that is, very low. Importantly, we discovered that the time elapsed between blood collection and processing has a massive effect on cytokine release that would easily mask any effect of a genetic variant. Using this system, we reveal a large effect of the rs17561 minor allele on IL‐1α release that reached statistical significance in a relatively small cohort of individuals.

Recent notable use of the whole blood stimulation system was in the assessment of typical healthy immune responses to a range of stimuli [16]. Duffy et al also reported robust IL‐1α release in response to LPS, along with heat‐killed *E. coli*, but unexpectedly found a subpopulation of individuals that failed to release any IL‐1α in response to any stimuli. These non‐responders occurred at ~8%, which is highly comparable to the frequency of rs17561 minor allele homozygotes (Figure 1d). Although our data clearly shows rs17561 minor allele homozygotes secrete less rather than no IL‐1α, we did also identify failed IL‐1α secretion within the cohort. Two non‐responders were witnessed out of fifty individuals, representing 4%, with both being rs17561 minor allele homozygotes. Thus, the possibility exists for rs17561 to be in linkage disequilibrium with another unidentified IL‐1α SNP that either synergises with rs17561 or entirely prevents IL‐1α secretion itself. However, significantly more individuals would be required to identify the true frequency of these non‐responders and if any association to rs17561 exists. Interestingly, rs1800587 is in linkage disequilibrium with rs17561 and is located within the *IL1A* promoter. However, we find no effect of rs17561/rs1800587 on *IL1A* or *IL1B* transcript level (Figure 5) (in keeping with a previous study [14]) and also find reduced secretion of the S^114^ minor allele form in macrophages (Figure 8a), validating that the rs17561 amino acid substitution directly mediates the effect. In addition, because the individuals failing to release IL‐1α secreted normal levels of IL‐1β, this further supports that IL‐1α and IL‐1β can be released independently from one another. Although we show more S^114^ IL‐1α is retained within activated macrophages (Figure 8c) (perhaps suggesting altered charge modifies intracellular binding), the specific mechanism of IL‐1α release still remains unknown, with a significant component caspase/inflammasome independent (Figure 7c). Although nuclear localisation can retain IL‐1α upon necrosis [11], the rs17561 substitution is 27 amino acids away from the NLS that controls import. Thus, we currently do not have a full biochemical explanation for why less S^114^ IL‐1α is released from cells.

The rs17561 minor allele is associated with both increased and decreased risk of disease (Table 1). The reduced IL‐1α secretion reported here concurs with the known aetiology of several diseases showing decreased risk. For example, nasal polyposis is driven by asthma‐associated chronic inflammation [29], while systemic sclerosis [30] is an autoimmune disease with a strong inflammatory component, and thus less IL‐1α would understandably benefit. However, the mechanism for reducing IL‐1α and increasing the risk for other diseases may initially seem counterintuitive. For example, in both breast [31] and ovarian cancer [32, 33] risks are increased by the rs17561 minor allele, suggesting more IL‐1α is beneficial. Yet, IL‐1α drives inflammation, which is one of the hallmarks of cancer, with chronic inflammation both initiating cancer via genotoxic stress, and driving tumorigenesis via cell proliferation, angiogenesis and tissue invasion [34]. In contrast, inflammation and innate immune activations are required for an adaptive immune response, and thus cancer immunosurveillance and tumour control. Similarly, IL‐1 drives effector T cell proliferation, even in the presence of Tregs, which could enable adaptive responses within an immunosuppressive tumour milieu. In addition, IL‐1α drives the SASP, which reinforces cellular senescence/cell cycle arrest, and thus acts as an important barrier to stop the malignant transformation [35, 36, 37]. Furthermore, the IL‐1α‐driven SASP also recruits and instructs immune cells to remove senescent cells (senescence surveillance) before bypass reinitiates tumorigenesis. Thus, IL‐1α‐driven processes can play a dual role during cancer, which is likely context‐dependent. Although we show the effect of the rs17561 minor allele in monocytes and macrophages, whether this also applies to non‐myeloid cells is currently unknown. One approach to investigate this would be to generate different cell lineages from iPSCs taken from major/minor homozygous individuals. Together, understanding cell type‐specific and disease‐specific effects of rs17561 might aid patient stratification in the use of anti‐IL‐1 therapies.

In conclusion, using a recall‐by‐genotype study we show that rs17561 minor allele homozygotes release significantly less IL‐1α than the major allele. Thus, rs17561 represents a hypomorphic mutation that can be used to determine the specific contribution of IL‐1α to disease and/or physiological processes.

## AUTHOR CONTRIBUTIONS

Kimberley A. Wiggins, Katerina Pyrillou and Melanie Humphry performed experiments, designed experiments and analysed data. Adam S. Butterworth analysed plasma proteomes for associations. Murray CH. Clarke conceived the project, designed experiments, analysed data and wrote the manuscript with Kimberley A. Wiggins and Katerina Pyrillou.

## CONFLICT OF INTEREST

All authors declare no conflicting interests, financial or otherwise.

## Supporting information


**Figure S1** The IL‐1α A^114^ residue is highly conserved across multiple species. Multi‐species alignment of IL‐1α amino acid sequences showing very high conservation of Ala or a conservative substitution to Val.

**Table S1** Diseases and conditions with a reported genetic association to the rs17561 minor allele. Published studies report an association between the rs17561 minor allele and disease, suggesting the non‐synonymous mutation in IL‐1α produces an amino acid substitution that alters function. n.b. earlier discovery GWAS studies may not have been fully replicated in later large BioBank studies.

**Table S2** Traits with a significant association to the rs17561 minor allele variant. Data from PheWAS and Gene ATLAS database showing beta, p value and odds ratio for traits associated with the minor allele variant of rs17561. n/a = not applicable/available.

## Data Availability

The data that support the findings of this study are available from the corresponding author upon reasonable request.
