# Supplementary figures and images for: The common IL1A single nucleotide polymorphism rs17561 is a hypomorphic mutation that significantly reduces interleukin‐1α release from human blood cells
Source: Immunology. 2022 Oct 13;168(3):459–72. doi: 10.1111/imm.13584 (PMC11495263; doi:10.1111/imm.13584)

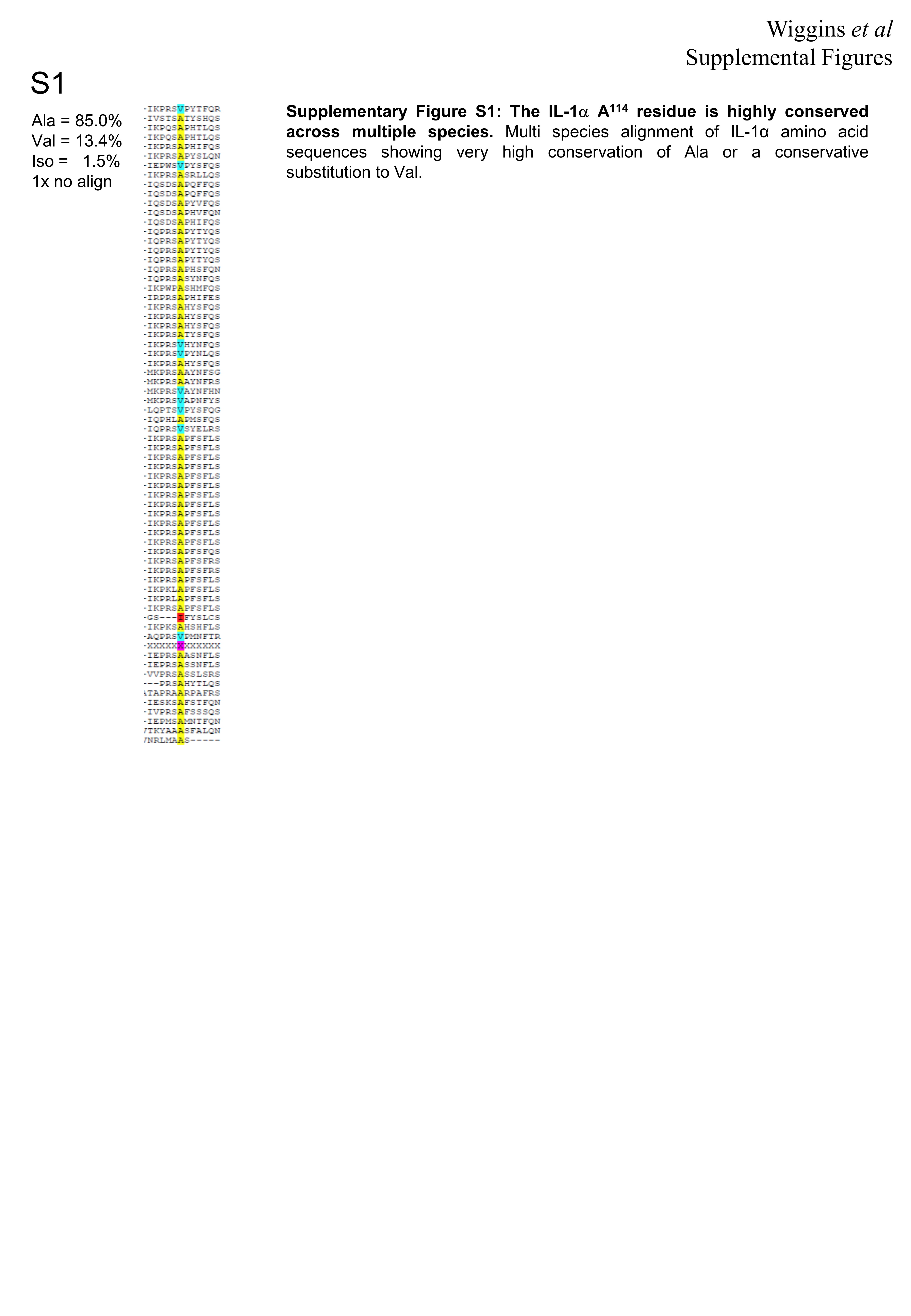

Supplement: Supplementary file 1 — Figure S1 The IL‐1α A114 residue is highly conserved across multiple species. Multi‐species alignment of IL‐1α amino acid sequences showing very high conservation of Ala or a conservative substitution to Val. [file IMM-168-459-s002.jpg]
