# Supplementary material for: The common IL1A single nucleotide polymorphism rs17561 is a hypomorphic mutation that significantly reduces interleukin‐1α release from human blood cells
Source: Immunology. 2022 Oct 13;168(3):459–72. doi: 10.1111/imm.13584 (PMC11495263; doi:10.1111/imm.13584)
Supplement: Supplementary file 2 — Table S1 Diseases and conditions with a reported genetic association to the rs17561 minor allele. Published studies report an association between the rs17561 minor allele and disease, suggesting the non‐synonymous mutation in IL‐1α produces an amino acid substitution that alters function. n.b. earlier discovery GWAS studies may not have been fully replicated in later large BioBank studies. [file IMM-168-459-s003.docx]

|  | ***DISEASE/CONDITION*** |  | ***EFFECT OF rs17561 MINOR ALLELE*** |  | ***REFERENCE*** |
| --- | --- | --- | --- | --- | --- |
|  | Nasal polyposis |  | Protected against development of nasal polyposis in asthmatics |  | (Karjalainen et al., 2003)^28^ |
|  | Systemic sclerosis |  | Protective against disease |  | (Kawaguchi et al., 2003)^29^ |
|  | Malaria |  | Predispose to disease but protective for severe symptoms |  | (Walley et al., 2004)^37^ |
|  | Breast cancer |  | Increased risk of disease |  | (Sigurdson et al., 2007)^30^ |
|  | Ankylosing spondylitis |  | Increased risk of disease |  | (Sims et al., 2008)^38^ |
|  | Juvenile dermatomyositis |  | Increased risk of disease |  | (Mamyrova et al., 2008)^39^ |
|  | Chronic rhinosinusitis |  | Increased risk of disease |  | (Endam et al., 2010)^40^ |
|  | Acne vulgaris |  | Increased risk of acne |  | (Szabo et al., 2010)^41^ |
|  | Radiation-induced toxicity |  | Increased risk of toxicity following treatment for non-small cell lung cancer |  | (Hildebrandt et al., 2010)^42^ |
|  | Obesity |  | Increased body mass in healthy women |  | (Um et al., 2011)^43^ |
|  | Multiple sclerosis |  | Associated with earlier disease onset |  | (Mirowska-Guzel et al., 2011)^44^ |
|  | Ovarian cancer |  | Decreased risk of clear cell, mucinous and endometrioid subtype |  | (White et al., 2012)^31^ (Charbonneau et al., 2014)^32^ |
|  | H1N1 influenza A virus |  | Increased risk of disease |  | (Liu et al., 2013)^45^ |
|  | Coronary artery disease and cardiovascular disease |  | Higher risk of disease as part of a composite genotype and stratification according to lipid levels |  | (Tsimikas et al., 2014)^46^ |
|  | Periodontitis |  | Increased risk of disease |  | (Yin et al., 2016)^47^ |
|  | Lumbar disc degeneration |  | Increased severity |  | (Perera et al., 2017)^48^ |
|  | Asthma |  | Increased severity |  | (Leal et al., 2018)^49^ |
|  | Body Mass Index |  | Increased BMI |  | (Mendoza-Carrera et al., 2019)^50^ |
