# Supplementary material for: The common IL1A single nucleotide polymorphism rs17561 is a hypomorphic mutation that significantly reduces interleukin‐1α release from human blood cells
Source: Immunology. 2022 Oct 13;168(3):459–72. doi: 10.1111/imm.13584 (PMC11495263; doi:10.1111/imm.13584)
Supplement: Supplementary file 3 — Table S2 Traits with a significant association to the rs17561 minor allele variant. Data from PheWAS and Gene ATLAS database showing beta, p value and odds ratio for traits associated with the minor allele variant of rs17561. n/a = not applicable/available. [file IMM-168-459-s001.docx]

|  | ***TRAIT*** |  | ***BETA*** |  | ***pVALUE*** |  | ***ODDS RATIO*** |
| --- | --- | --- | --- | --- | --- | --- | --- |
|  | Triglyceride level |  | -0.01232 |  | 0.000003 |  | n/a |
|  | Mean platelet volume |  | 0.00588 |  | 0.000692 |  | n/a |
|  | Platelet distribution width |  | 0.00370 |  | 0.000087 |  | n/a |
|  | Reticulocyte % |  | 0.00285 |  | 0.006943 |  | n/a |
|  | ….. |  |  |  |  |  |  |
|  | Dysmenorrheic pain severity |  | 0.51280 |  | 1.36x10^-11^ |  | n/a |
|  | High blood pressure |  | -0.00496 |  | 5.80x10^-7^ |  | n/a |
|  | Schizotypal disorders |  | -0.25800 |  | 0.000098 |  | n/a |
|  | Clear cell ovarian cancer |  | -0.14880 |  | 0.000751 |  | n/a |
|  | BMI |  | 0.02909 |  | 0.000966 |  | n/a |
|  | Rosacea |  | 0.00029 |  | 0.001223 |  | 1.21 |
|  | Ishaemic heart diseases |  | 0.00184 |  | 0.001136 |  | 1.03 |
|  | ….. |  |  |  |  |  |  |
|  | eQTL for LncRNA-CKAP2L |  | -0.25990 |  | 1.65x10^-92^ |  | n/a |
|  | eQTL for SLC20A1 |  | -0.15050 |  | 1.29x10^-31^ |  | n/a |
|  | eQTL for MERTK |  | -0.13014 |  | 5.31x10^-24^ |  | n/a |
|  | eQTL for PAX8 |  | 0.08206 |  | 2.05x10^-10^ |  | n/a |
|  | eQTL for IL-1RN |  | 0.08057 |  | 4.34x10^-10^ |  | n/a |
